# Developmental Changes in the Philippine Health System: Accomplishments, Successes and Challenges

**DOI:** 10.3390/healthcare7040116

**Published:** 2019-10-14

**Authors:** Xerxes Seposo

**Affiliations:** 1 School of Tropical Medicine and Global Health, Nagasaki University, Nagasaki 852-8523, Japan; seposo.xerxestesoro@nagasaki-u.ac.jp; 2 Faculty of Management and Development Studies, University of the Philippines Open University, Los Banos, Laguna 4031, Philippines

**Keywords:** Philippines, health systems, health determinant, health financing, health management

## Abstract

The Philippine health system has undergone various changes which addressed the needs of the time. These changes were reflected in the benchmarks and indicators of performance of the whole health system. To understand how these changes affected the health system (HS), this study determined the changes in the Philippine health system in relation to different health domains (health determinants, financing, and management/development). Two HS periods were identified, namely, health system period 1 (HS 1) from 1997–2007 and health system period 2 (HS 2) from 2008–2017. Each HS period was assessed based on three domains. The first two domains were quantitatively assessed based on an interrupted time-series method, while the third one underwent a comparative analysis using two Health Systems in Transition reports (2011 and 2018). This study was able to assess the developmental changes in the Philippine health system. Specifically, the (health determinant) maternal mortality rate (MMR) significantly decreased by three maternal deaths per 100,000 live births, the (health financing) tobacco excise tax increased by 13,855 (in Million PhP) in HS 2, and there was (health management/development) an improvement in access to health facilities. However, there was an indication of retrogressive progress with some challenges in HS 1 which remained unaddressed in HS 2. While it seems promising that the health system has progressed with improvements apparent in both health outcomes (e.g., MMR) and health financing (e.g., tobacco excise tax), such improvements were overshadowed by the inefficiencies, which were not addressed by the current health system (HS 2), thus making it more retrogressive than progressive.

## 1. Introduction

Health systems are structurally complex frameworks of intertwined operations which serve as a means in achieving pre-defined health goals set by an institution [1]. Health systems exist to complement other sectoral systems but are primarily dedicated in addressing the need of health care. Earlier records of existing health care systems were from the 600 BC where Charodes ordered that all citizens of Athens will have the right to access free medical care [2]. These so-called health care systems were not entirely “structurally systematic”, instead, these were unorganized and selective efforts to highlight the importance of (individual) health. Since then, various governments have engineered to create health systems which would go beyond personal/individual health to a broader societal breadth. Decades and centuries of development led to major health care system movements centered in Europe (UK), Asia (China), and North America (United States).

The National Health Service (NHS) of the UK was established in 1948 to render health care services at the national level coverage [3]. Its primary focus from 1950 to 1970 was on the modernization of facilities and technologies for improved health care delivery services. On the other side of the world, in China, since the founding of the People’s Republic in 1949, Meng et al. [4] emphasized that the “formation and development of the health system, especially the organization and governance structure, have been closely related to political, economic and administrative reforms”. The main mover in the changes in China’s health system was mainly due to the country’s economic pursuits which lasted for more than 30 years [4]. In the 1980s, the implementation of China’s Open Door Policy has allowed an influx of foreign investments, which then allowed the country to bolster its health financing schemes, which are linked to the economic developments brought forth by the policy [5]. Then there is the US health system, which began in the private sector and took flight in the 1960s as a state-regulated program [6]. The health system founded from the private sector’s perspective had a domino effect on the rise of the private insurance system. Private health insurance in the United States had its beginnings around the early 1930s, with the establishment of non-profit Blue Cross plans for hospital care and soon after Blue Shield plans for physician care [6,7]. Whereas in the Philippines, the earliest concept of public health was introduced by the Franciscan Friars in 1577 [8]. Various developments with respect to health have been fostered by the previous colonizers. The Spaniards instituted the Superior Board of Health and Charity in 1888, whereas the Americans helped in the installation of a more formal health administration through the (1) Act 1507 of the Philippine Commission in 1901 setting up the Board of Health of the Philippine Islands, and (2) Act 307 through 309, which provided provincial and municipal boards for health [8]. Forty years later, the Philippines independently created its own health system in 1941, where the Department of Health was separated from the Department of Health and Public Welfare and was established as a separate entity [9]. The Philippine health system operates in a devolved manner owing to the local government code of 1991, whereby services were mostly under the jurisdiction of the local governments, with supplementary services such as major national programs, which include but are not limited to immunization, tuberculosis, nutrition, etc. Decentralization of health services became a center piece of the Philippine health system.

Each of these health systems have been tailor-fitted for the specific situation for each country. The Philippines’, in particular, is unique from other major countries’ health systems as it is one of the few archipelagic country health systems. The Philippines’ health system provides an opportunity for examining the pertinent health system challenges geographically diversified countries experience. Likewise, as health systems change through time, temporal developmental changes though apparent, have not been elucidated and their assessment remains a challenge. While there have been numerous legislations which introduced changes in the Philippine health system [10], most of the major changes happened from or took effect relatively near to 2008. These health system-related changes included the universal access to cheaper and quality medicines (in 2008), FOURmula One for Health, an operational framework for health reform agenda which started from 2005 but gained momentum in 2007, and inception of the Philippine Facility Enhancement Program in 2008, among others [10]. With these major developments, it is thus important to understand the developmental changes in the Philippine health system, particularly focusing on the past decades’ health systems’ (Health System 1: 1997–2007 versus Health System 2: 2008–2017) accomplishments, successes, and challenges. Specific assessment of the developmental changes between Health System 1 (HS 1) and Health System 2 (HS 2) in terms of three domains—namely, (a) health determinant, (b) health financing, and (c) health development/management—were carried out.

## 2. Materials and Methods

The health system goals highlighted in the WHO Framework for Health System Performance Assessment [11]—namely, (a) health, (b) fair financing and financial risk protection, and (c) responsiveness—were operationalized into the three domains in this study, which were (1) health determinant, (2) health financing, and (3) health management/development. Murray, et al. [11] emphasize the monitoring of these three intrinsic goals as a main basis for health system performance. In brief, the first goal regarding health anchors to the prime motivation of a health system, and that is to improve the health of the population. This can be represented by a non-exhaustive list of health determinants, which include the incidence of a disease in the population, life expectancy, and mortality rates. The second goal focuses on the financial aspect of the health system, both in the household and society levels. This can be represented by the household level contribution to the health system or funding streams coming from either internal tax revenues or external donor aids. Lastly, responsiveness tackles the expectations of the population regarding the health system catering to the population’s needs. The health system responds to the population’s needs via provision of services and is closely linked to health administration/management. Addressing previously encountered health system related issues, through improved health management and administration, constitute health system responsiveness. In this study, the variables representing the health determinants and health financing were selected based on whether they satisfy the conceptual definitions of health system goals of “health” and “fair financing and financial risk protection” as highlighted in the WHO Framework for Health System Performance Assessment, and if they have existing annual data.

**Figure 1:**
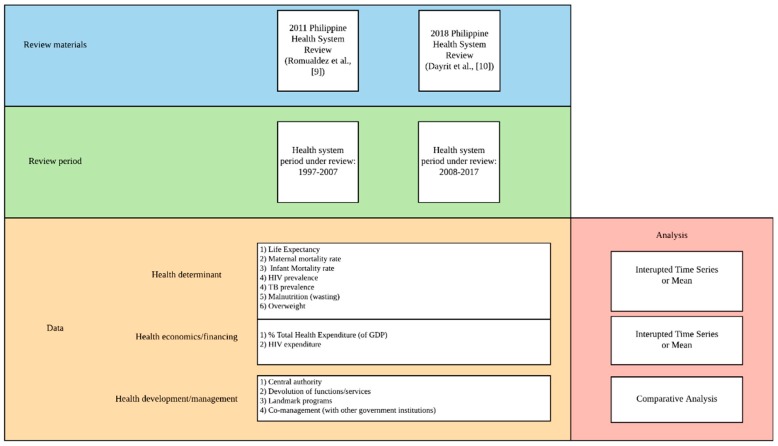
Health system analysis methodological framework.

Each of the domains were analyzed separately with the respective data and methods to be used, as shown in Figure 1. A mixed method was utilized to analyze each domain, quantitative for health determinant and health financing, whereas qualitative for health management/development. Data used in the respective analyses are summarized in Supplementary Materials Table S1.

### 2.1. Data Description

#### 2.1.1. Health Determinant Domain

Health determinant data, such as life expectancy at birth, maternal mortality rate, infant mortality rate, HIV incidence, TB incidence, severe wasting, and overweight, were obtained from online data sources, as shown in Table S1 with the corresponding variable description and source summarized in Table S2. While there are numerous candidates for health determinants (e.g., cancer and other chronic diseases), due to the annual data unavailability which could match the time periods, only the aforementioned health determinant data were analyzed.

#### 2.1.2. Health Financing Domain

Data for health financing were obtained from the World Bank (WB) database. Total health expenditure in terms of percentage of GDP (%GDP) was extracted from the WB world development indicators database [12]. While there may be other variables which could represent total health expenditure, Mcintyre, et al. [13] argue that the “percentage of GDP provides a basis in advocating an in increase in both government resource mobilization and spending on the full range of human rights and social determinants of health in situations where governments are not presently providing maximum available resources”.

#### 2.1.3. Health System Management/Development Domain

Main data source for analyses included the grey literature of the Health Systems in Transition (HIT) reports (2011 and 2018 versions) of the Philippine Health System. In support to the HIT reports are the NHA reports, as well as Philippine Institute of Development Studies (PIDS) reports, together with some relevant legal statutes (republic act and implementing rules and regulations) passed or took effect during the specific periods.

## 3. Analyses

### 3.1. Interrupted Time Series

Data from the health determinant and health financing domains were analyzed using an interrupted time-series (ITS) technique. This is a quasi-experimental design, which aims to evaluate the impact of interventions in a longitudinal framework of observations [14,15]. Interrupted time series has been gaining traction and has been implemented in health care studies which assess the attribution of the changes an intervention has introduced compared to a no-intervention scenario [14,15,16]. It is characterized by the temporal, time-related changes through the difference in the trend of pre- and post-periods of interest. In this case, the pre-period was the 1997–2007 health system, while the post-period was 2008–2017. The selection of the division of the periods was based on the major changes which occurred starting from or relatively near to 2008, as highlighted in Dayrit et al. [10]. In this study, 2008 was assumed as a pivotal point where major changes after 1991 were introduced to the health system. The proposition is, if the changes implemented in or relatively near to 2008 had not happened, would the health system, in terms of the health determinant, health financing, and health management components, remain the same? In effect, there were two health system periods, before and after the major changes. We then used this division to assess progress in the health system.

**Figure 2:**
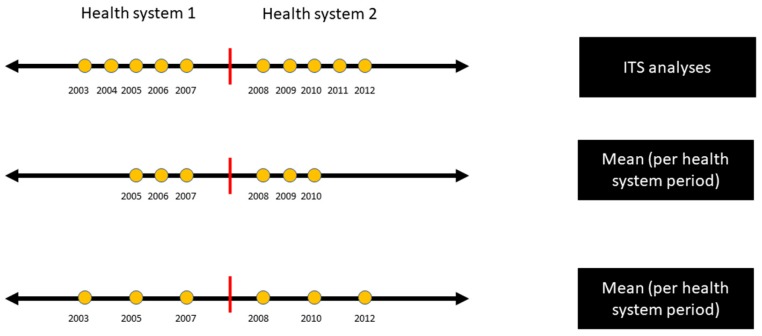
Analysis strategies subject to the availability of the data points for each variable. ITS = interrupted time-series.

The ITS was parameterized as in Equation (1) below:

(1) $$y=\beta_{0}+\beta_{1}T+\beta_{2}par+\beta_{2}par:T+\varepsilon$$

whereby, y is the parameter of interest (either health determinant or health financing domain variables), T is the time component, whereas *par* is the binary variable indicating the 1997–2007 (valued to be 0) and 2008–2017 (valued to be 1) health systems; ε is the error term representing the uncertainty in the model [17]. The cut off period for the ITS was set at 1997/1998–2007 and 2008–2017/2018. At least five continuous annual data points should be available for the ITS analysis. If the number of continuous data points for each period was less than five or if the data points were not continuous, the mean of the data points was utilized instead; as shown in Figure 2 below.

While the minimum number of observations to carry out ITS was 3 [18], in this study, variables with at least 5 temporally consecutive observations before and after 2008 were analyzed (for ITS). On the other hand, the mean was taken for variables which were either (a) non-consecutive 5 observations or (b) less than 5 observations.

The variables/parameters subject to ITS analyses were:

(a) Life expectancy;
(b) Maternal mortality rate;
(c) Infant mortality rate;
(d) HIV prevalence;
(e) TB prevalence;
(f) Total health expenditure;
(g) Tobacco excise taxes.

While the following variables were averaged for each health system:

(a) Malnutrition (wasting);
(b) Overweight;
(c) HIV expenditure.

### 3.2. Comparative Analysis

To facilitate the health system management/development analysis, a comparative analysis was conducted utilizing the papers of Romualdez et al. [9] and Dayrit et al. [10], which represented health system period 1 (HS 1) from 1997–2007 and health system period 2 (HS 2) from 2008–2017, respectively. Each of the reports were summarized into four components, namely, (a) Feature, (b) Challenges, (c) Reforms, and (d) Laws/Statutes passed/enforced (during the said period). “Feature” reflects the characteristics inherent to the health system, with ample focus on the strengths. “Challenge” are the health issues or problems arising under the HS period. “Reforms” are the innovations introduced in the said HS, and “Laws” are landmark statutes passed/enforced.

**Figure 3:**
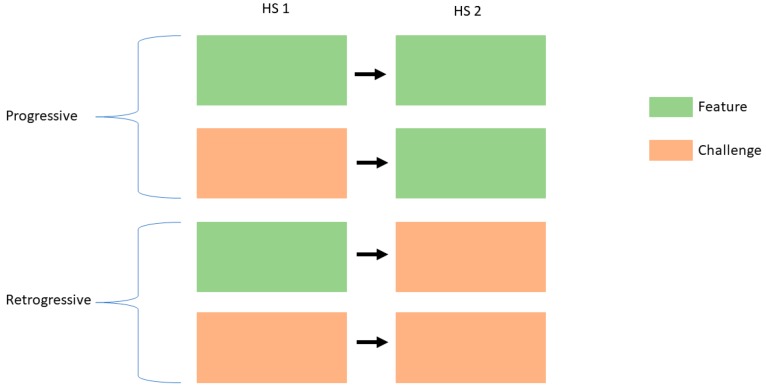
Graphical summary of health system development.

After summarizing the report into these four components, only the “Feature” and “Challenge” components were utilized to represent health system development. In this study, HS development was defined in terms of being (a) Progressive or (b) Retrogressive using matched variables from the HS 1 and HS 2. Progressive classification was defined in terms of two aspects: (a) Challenge changed to Feature; (b) Feature retained as Feature. When a challenge in HS 1 becomes a feature of HS 2, it means that HS 2 was able to progressively address the challenge. While, if the feature remains the same between two health systems, it only signifies that HS 2 was able to maintain that current feature. The HS 2 was retrogressive if a feature in HS 1 became a challenge in HS 2. Similarly, if a challenge in HS 1 remained to be a challenge in HS 2, it only indicated that there was not much progress in addressing such a challenge. A graphical summary is shown in Figure 3.

## 4. Results

From the health determinants, only TB prevalence had an apparent distinction among the two health system periods, with an apparent decrease in the previous one and a subsequent increase in TB cases in the succeeding health system period. There was no distinct trend for maternal mortality rate, while there was decreasing and increasing trends for infant mortality rate and HIV prevalence, respectively. All the data utilized in this study are reflected in Table S1.

All three health financing parameters increased with time, with an apparent and extensive increase with the excise tax from tobacco, representing the tobacco excise tax measures.

In Table 1, there was no significant change in life expectancy in either of the HS. While we observed relatively positive changes in HS 2, such as a statistically significant decrease in Maternal Mortality Rate (MMR) (a decrease of approximately three maternal deaths per 100,000 live births) and an increase in excise tax funds, a majority of health determinants such as infant mortality rate, HIV incidence, and TB incidence increased coupled with a decreased THE.

**Figure 4:**
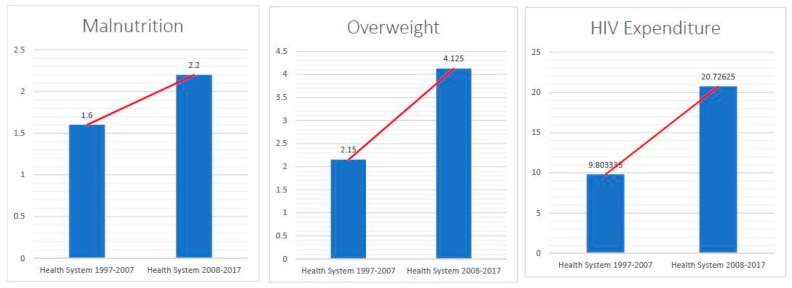
Malnutrition: Prevalence of severe wasting, weight for height (% of children under 5); overweight: prevalence of overweight, weight for height (% of children under 5); HIV expenditure: inflation-adjusted HIV expenditure (in USD millions); mean representation of the selected health determinant (malnutrition and overweight), and health financing (inflation-adjusted HIV expenditure) variables.

In Figure 4, we can observe that both malnutrition and overweight population increased by 37.5% and 91.9%, respectively. Both of which signify that non-communicable diseases have been on the rise in the Philippines, which may indicate some levels of underperformance in HS 2. We can find a rather interesting trend, in which though HIV expenditure, through both the local funds and foreign aids, increased significantly in HS 2, we still see an increasing trend of HIV incidence in the same period—a possible indication of either underperformance in disease prevention or an increase in detection rate. 

## 5. Discussion

This study was able to assess the developmental changes in the Philippine health system in the past decade. While there was progress in different health system components from the HS 1 to HS 2, the overall health system performance was retrogressive with major challenges still being carried over to HS 2.

In Table 1, there was a significant decrease in the number maternal deaths in HS 2 than in HS 1. However, all other health determinants were found to be retrogressive. In the case of health financing, while THE decreased in HS 2, other health financing variables increased. The tobacco excise tax significantly increased coupled with the doubling of HIV expenditure in the past decade. As for the health system management/development, though there were some progressive changes in the health systems (increase in health financing, increase in health facility access, increase in social health insurance coverage, and improved waiting time). Taking all developmental changes into account, overall, the health system is still facing the challenge of retrogressive progress.

While it seems promising that the health system has progressed with improvements apparent in both health outcome (i.e., MMR) and health financing aspect (i.e., tobacco excise tax), such improvements were overshadowed by the overwhelming inefficiencies which were not addressed by the current health system, thus leading it to be retrogressive rather than progressive.

**Figure 5:**
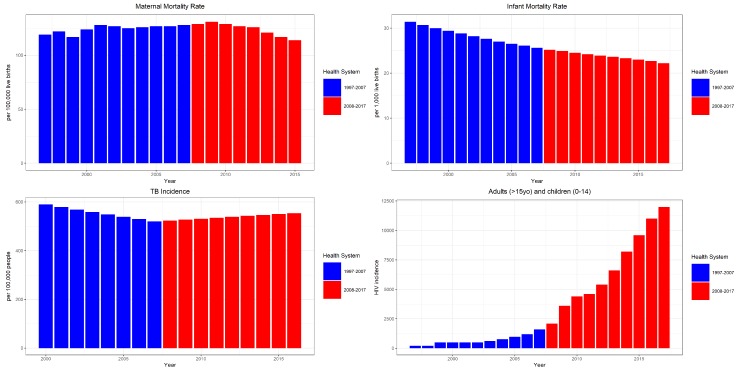
Health determinants in various health systems.

**Figure 6:**
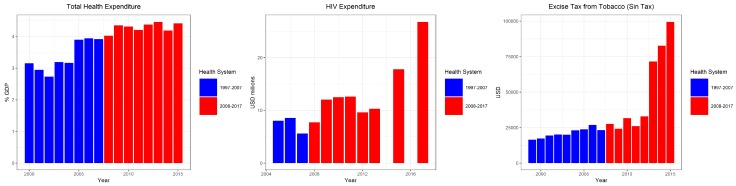
Health financing parameters (total health expenditure, HIV expenditure, tobacco excise tax) in either of the health system periods.

The Philippine health system has progressed in one way or another, but there are some developmental aspects which remained unaddressed. Utilizing the whole period data, we can observe only a few variables which indicate a definite trend. In Figure 5, IMR is gradually decreasing, whereas HIV incidence has increased rapidly through the years. In health financing, as shown in Figure 6, all variables increased through the years. However, Figure 4 and Figure 5 may not be enough to understand how the health system progressed amidst the various changes it had undergone through time, particularly with the major changes in 2008. The succeeding sub-headings focus on how the health system changed (comparing HS 1 and HS 2) based on the premises of: (a) health determinants, (b) health economics/financing, and (c) health system management/development.

### 5.1. On Health Determinants

Among the health determinants, only MMR significantly progressed in HS 2 compared to HS 1. The progress in MMR can be attributed to the improvement in the health service facilities as well as the increase in access to health services highlighted in Table 2 (HS 2: Feature 9). Reforms such as the HFEP and the DOH deployment program (in HS 2) are essential components in health service accessibility in the rural areas. All other health determinants increased in HS 2. The IMR increased, with approximately three infant deaths per 10,000 live births, there were 965 new HIV positive cases, and 14 new TB cases in HS 2 (shown in Table 1).

The summary table from the health systems in transition reports of Romualdez et al. [9] and Dayrit et al. [10] were outlined in four aspects, namely, (a) Features, (b) Challenges, (c) Reforms, and (d) health-related Laws accompanying or independent of the reforms. Changes in the health system were more apparent in HS 2 with major features of health insurance and health facility reforms. However, such features were also accompanied with challenges related to OOP and NCD-related health problems.

While there were health reforms in the past HS, IMR progress may have stagnated due to the presence of socioeconomic factors in relation to within-country inequalities in child health outcomes [19]. Kraft et al. [19] noted that child mortality in the Philippines may vary in terms of the rural–urban divide, by province and wealth status. Implementation of these reforms toward the grassroots level may have been trickling down at a different pace, thus an inequality in terms of the health outcomes in the provinces exists. This would have later impacts on the national level progress in IMR. This is further supported by the “Challenges” observed in HS 2 in Table 2, whereby there was an uneven distribution of health facilities and personnel across the country.

The increasing TB incidence in the country may be related to either (a) the inefficiency of health interventions in reaching the risk populations or (b) the detection of cases was enhanced. According to the GBD Tuberculosis Collaboration Group [20], the little or non-improvement in the TB situation in the Philippines has been linked to the inadequacy of the diagnostic test and its use in the risk populations (prison inmates and indigenous populations). This is further worsened by the increasing prevalence of multidrug-resistant TB and extensively drug-resistant TB in the country [21] as well as the increasing HIV incidence. The link between TB and HIV has been well established, with HIV infection increasing the risk by 20 times compared to HIV-seronegative individuals in highly prevalent countries [22]. From 1990 to 2003, HIV infection is one of the key underlying factors for the 1% increase in annual global TB incidence [23]. On the other hand, the increase in TB incidence may also be explained by the increase in the detection capacity. Vianzon et al. [24] highlighted that the increase in TB incidence can be attributed to the initiatives of the National TB Program in improving access to diagnostic and treatment services. Public–private partnerships have also contributed to the increase in the detection rate of TB incidence [25].

Also, HIV was observed to have increased in HS 2, similar to TB. This may be viewed as an inefficiency of the health services in reaching at risk populations, otherwise, it may be an efficiency in the detection rate. There was a significant increase in HIV incidence, with 965 new cases higher—than in HS 1. Some attributed the increase of HIV incidence to the drug-resistant subtype of HIV, specifically the change in the typically observed Western subtype B to a more aggressive HIV subtype, AE [26]. However, from the health management side, Farr and Wilson [27] identified that low rates of condom use, increased casual sex activities, and the widespread misconception about HIV/AIDS has driven the disease into becoming an epidemic. The stigma regarding HIV/AIDS has been acknowledged in HS 2 (Table 2: Challenge 11) but has not been fully addressed in HS 1. Though there were problems regarding HIV/AIDS in HS 1, there was no explicit mention of the stigma problems faced by the key populations. The lack of acknowledgement about the stigma may be due to the less attention it gained during HS 1, and when the epidemic exponentially increased after 2008, it gained more attention. On the other hand, the increasing HIV incidence may be related to the increase in the awareness and access to health services [28]. While this is plausible, the continuous and rapidly increasing rate of HIV incidence may prove to be more than just the increase in access/awareness; rather, this may be rooted in a more complex interaction of the previously highlighted molecular and management factors.

Aside from IMR, TB, and HIV increases, malnutrition and overweight increased by 37.5% and 91.9%, respectively (shown in Figure 4; leftmost and center panels). Nutrition-related problems are precursors to even bigger problems in the Philippines—non-communicable diseases (NCDs). These constitute 57% of annual deaths in the country, and had been increasing over the last decades [29]. Adair [30] noted that there was a six-fold increase of overweight and obese women from a 6% in 1983–1984 to a 35% in 1998–1999. The increase in these nutrition-related problems, particularly overweight, can be explained by the change in food regimens, whereby there is greater consumption of high caloric and less essential nutrient food sources and is further worsened by the sedentary life style [30].

### 5.2. On Health Financing

An efficient health financing would promote overall health and improve health status. While the tobacco excise tax was statistically higher in HS 2, at 13,855 (in Million PhP) and THE decreased by 0.135. The increase in the tobacco excise tax has been linked to improvements in personal health and eventually for the health system as a whole in the long run. In Lebanon, Salti et al. [31] observed that the tobacco tax resulted in an estimated 65,000 averted premature deaths, 300 million USD additional tax revenues, and 23 million USD out-of-pocket spending on health care averted. Both the financial and health benefits of increasing tobacco taxes result in health gains and savings in health care, which could influence later on improvement in the health system. In China, Verguet et al. [32] had similar findings wherein an increase in tobacco tax resulted in a pro-poor policy instrument bringing health and financial benefits to households and substantial revenues to society. The HIV expenditure (Figure 4; rightmost panel) paired with the findings about increased incidence can be interpreted in two ways: (a) inefficient resource use and (b) increase in detection. Though it increased by 82.3% in HS 2, new HIV cases surged in HS 2, which is quite counterintuitive. This may be linked to inefficient resource use, particularly in using the current resources to decrease the incidence. On the other hand, it could mean that resources may have been used to increase detection of new cases. If it is the first one, then this seemingly increasing resource allocation to the HIV agenda can be an opportunity to look back and reevaluate resource efficiency subject to the targets and goals set. There were various factors linked to the worsening HIV epidemic, highlighted previously, and these factors can be candidate starting points, which can be utilized in re-strategizing how to maximize the use of resources in achieving the desirable output, outcome, and impact of interest. While if it is the other way around, there is a need to assess the scale of the operations in what could be the most efficient way to improve current practice.

### 5.3. On Health Management/Development

The aim of assessing the health management/development was not to judge which health system is better, rather, if the health system, divided into two periods, was able to progress amidst the challenges and reforms it has undergone. Both HS 1 and HS 2 had their unique features setting them apart (as summarized in Table 2). The HS 1 was the precursor of primary health care and social protection, and these features were further carried over to HS 2, with improvements in health facilities and health care benefits. In this study, progress was defined in terms of (a) when a feature in HS 1 was still existing in HS 2 (meaning it was sustained) and (b) when a challenge in HS 1 became a feature in HS 2 (means the problem was resolved and became an asset in HS 2). On the other hand, retrogression was defined as (a) when challenges in HS 1 were still existing in HS 2 (unresolved previous problems) and (b) when features in HS 1 became a challenge in HS 2 (an asset becoming a problem). Although there were numerous challenges and features (in Table 2), only those matched ones were included in the assessment in Table 3. Progressive aspects of the health system were apparent with the increased health financing, social protection, improved waiting time, and improved access to services and data gathering. However, a big portion of these developments were retrogressive, such as high OOP, uneven distribution of health facilities and personnel, and restrictions to health data access, among others. The retrogressive nature of development of the Philippine health system is related to the unresolved previous problems, partly due to the increasing challenges and limited resources to resolve existing ones. Even though investments in health are increasing, so too are the problems, thus these resources are spread thinly creating relatively small impacts on massive problems, resulting in these problems being unresolved. Constant evaluation of these programs and projects is needed to determine the evolving problem, and how to provide a way which adapts with the evolving problem. This is where information and data play a big role. This challenge has been acknowledged in both HS 1 and HS 2. The private health sector constitutes a large proportion of the health system and yet there is no access to the information they hold. Similarly, the public health sector has a weak data gathering, updating, and validation system, which makes health policy stationary and creates non-adapting solutions to evolving problems. If we have access to this information, we would have a clearer picture of where we are now, and how to move forward in the health system.

Progressive classification was defined in terms of two aspects: (a) Challenge changed to Feature; (b) Feature retained as Feature. When a challenge in HS 1 becomes a feature of HS 2, it meant that HS 2 was able to progressively address the challenge. While, if the feature remained the same among the two health systems, it only signified that HS 2 was able to maintain that current feature. The HS 2 was retrogressive if a feature in HS 1 became a challenge in HS 2. Similarly, if a challenge in HS 1 remained a challenge in HS 2, it only indicated that there was not much progress in addressing such a challenge. While there were important aspects of progress in the health system, it was still overwhelmed with challenges which were yet to be addressed. Health financing seemed to have improved; however, health insurance claims were stagnating and was worsened with the increase in or reliance on OOPs for health services.

## 6. Limitations

The study had a few limitations, namely, (a) availability of current data, (b) the number of observations for ITS, and (c) qualitative analysis.

There are various variables which can represent the health determinants, health financing, and health management/development domains; however, due to the data limitations, variables which were readily available or could be extracted were only used. Other health determinants data such as cancer rates and chronic health outcomes, as well as health financing data (e.g., catastrophic health spending), could also provide insights in regard to the performance of the health system. Future studies are encouraged to include these variables if available.

For health management/development, there may be other features and challenges not fully captured in the summary; however, if such information becomes available in the near future, there is a need to run a re-analysis. While the current number of observations before and after 2008 were beyond the minimum number of observations required to do an ITS [18], extending the number of observations may provide a more robust insight on the estimates. In this study, the quantitative analyses relied on the available data sources as well as grey literature in assessing the progress of the Philippine Health System. While the results of the quantitative techniques complemented the observations from the health transition reports, more detailed insights could have been gained from in-depth qualitative analyses through focus group discussions (FGDs) and key informant interviews (KIIs).Focus group discussions and KIIs would be able to provide a grassroots perspective of how the progress affected the beneficiaries, the people, as well as various insights on how progress was optimized and the challenges were resolved from a health manager’s perspective. Aside from FGDs and KIIs, the assessment of quality of life as well as patient satisfaction would provide additional qualitative evidence in support of the performance of the health system [33]. Further studies including qualitative analyses are needed to gain more insight into how health system progress is appraised from a community level perspective.

## 7. Conclusions

This study assessed the developmental changes in the Philippine health system, particularly focusing on the past decades’ accomplishments, successes, and challenges. The health system has progressed, particularly in the improvement of health outcomes (i.e., MMR), increased health financing, and increase in access to health services. However, there are indications that the overall health system is retrogressive. There are a multitude of factors which led to a retrogressive status. Similarly, there are different ways to address these issues, one of which is to constantly monitor and evaluate under the premise that data should be shared across stakeholders sanctioned by equivalently strict and secure data sharing/privacy agreements.

## Figures and Tables

**Table 1:** Interrupted time series results for each variable of interest.

Health Domain	Variables	Estimate	Standard Error	*p*-Value
Health determinants	Life Expectancy	−0.007	0.005	-
	Maternal Mortality Rate (MMR)	−3.25	0.435	***
	Infant Mortality Rate (IMR)	0.260	0.015	***
	HIV Incidence	965	57.5	***
	TB Incidence	13.7	0.086	***
Health financing	Total Health Expenditure (THE)	−0.135	0.048	**
	Tobacco Excise Taxes	13855	2824	***

*p*-value: *** *p* < 0.001; ** 0.001 < *p* ≤ 0.05; - statistically not significant.

**Table 2:** Summary table of the Features, Challenges, Reforms, and the pertinent Laws/Statutes passed and enforced during the respective health systems.

	Health System 1 (1997–2007)	Health System 2 (2008–2017)
Features	1. Decreasing Maternal Mortality Rate (MMR)/Infant Mortality Rate (IMR)	1. Expansion of PhilHealth coverage; however, low financial protection
	2. Private health sector constituted bigger proportion in health service delivery than public health sector	2. Data gathering was existent; however, intensified and modernized effort is needed
	3. Decentralization of health care services (fragmented health service delivery)	3. Intersectoral approaches to health and in its investment programming at the national and local levels (unified targeting for poor, etc.)
	4. Emphasis on primary health care	4. Increase in client satisfaction to government health services
	5. Rapid increase in nursing schools	5. Concerted efforts to ensure health care data privacy
	6. Introduction of health technology assessment (HTA) by PhilHealth (in identifying priority problems on the use of medical technologies needing systematic assessment)	6. Care-seeking behavior was dictated by ability to pay
	7. Increasing PhilHealth coverage	7. Waiting time improved
	8. Waiting time/hospital length of stay decreased	8. Treatment seeking attitude improved among households
	9. Migration of health workers, particularly nurses	9. Increased use of rural health units, decrease use of private clinics
	10. Increase in health financing	10. MMR decreased due to the increased facility-based deliveries and skilled birth attendants
		11. Existence of palliative care (cancer patients)
		12. Success in closing the gender gap
		13. Disaster health management system in place
		14. Increase in health financing
Challenges	1. Rising non-communicable diseases (NCDs)	1. Problems with devolved health financing and service delivery (fragmented strategy)
	2. High cost of accessing health service	2. Uneven distribution of health staff across the country (concentrated in National Capital Region)
	3. Low level financial protection	3. Uneven distribution of health facilities across the country (concentrated in NCR)
	4. High out-of-pocket (OOP) payments	4. TB Directly Observed Treatment Short Course (DOTS) accreditation is low
	5. Absence of an integrated curative and preventive network	5. Overregulation of programs (National TB Program and PhilHealth)
	6. Weak health information system/governance	6. High OOP payments
	7. Absence/lack of access of private sector data	7. Even though health services were utilized, this did not directly translate to health status improvement
	8. PhilHealth still used paper-based claims management	8. PhilHealth insurance claims stagnated at 33%
	9. Lack of health service information (PhilHealth)	9. Hospital bed availability was a difficulty
	10. Weak/non-existent structures in engaging community and patient participation with regard to health decision-making	10. Geographical constraints in service delivery (geographically isolated and disadvantaged areas)
	11. Members’ perceptions are that they have insufficient information and that the transactional requirements to make claims were too large	11. Stigma (HIV) and self-stigma (TB) were major barriers to care
	12. Low sponsored program PhilHealth utilization rate	12. Obesogenic environment; life-style related health problems
	13. Uneven distribution of PhilHealth accredited providers (35% of doctors are in NCR)	13. Air pollution and household air pollution
	14. Uneven distribution of health facilities and beds across the country	14. Low childhood immunization due to the fact of religious/cultural beliefs, as well as lack of coordination among public sector
	15. Lack of geriatric facilities and services	15. Healthcare provision tended to be either underprovided or overprovided, and costly
		16. Adherence to clinical practice guidelines were loose
		17. Patient safety data was lacking
		18. Health equity issues included the apparent urban–rural divide
		19. Health technology assessment (HTA) was yet to be fully established
		20. Health data acquisition was still restricted (private sector, public sector, PhilHealth)
		21. Fragmented nature of health financing, devolved structure of service delivery, and mixed public–private health system posed immense challenges in monitoring health sector performance
		22. Issues with conflict of interest (physician-owned pharmacy)
Reforms	1. Primary health care focus	1. Primary health care expansion due to the intensified HFEP
	2. Health Facility Enhancement Program (HFEP)	2. Deployment programs of the DOH and Local Government Units (LGUs)
	3. Health sector reform agenda (HSRA) launched	
	4. Corporatization of hospitals under HSRA	
Health-related laws accompanying or independent of the reforms	1. Republic Act No. 8344 “An Act Prohibiting the Demand of Deposits or Advance Payments for the Confinement or Treatment of Patients in Hospitals and Medical Clinics in Certain Cases”	1. Sin Tax Law of 2014
	2. Republic Act No. 7305 “Magna Carta for Public Health Workers”	2. National Health Insurance Act of 2013
	3. Republic Act No. 9184 “Government Procurement Reform Act”	3. Reproductive Health Law of 2012
	4. National Health Insurance Act of 1995 amended to Republic Act No. 9241	4. Tuberculosis Law of 2016
	5. 1988 Generics Act, amended to Republic Act No. 9502 “Cheaper and Quality Medicines Act”	

NCR = National Capital Region.

**Table 3:** Highlighting the health system development from HS 1 to HS 2 using matched variables.

Health System Development	Health System 1 (1997–2007)	Health System 2 (2008–2017)
Progressive	Increase in Health Financing	Increase in Health Financing
	Increasing PhilHealth coverage	Expansion of PhilHealth coverage; however, low financial protection
	Waiting time/hospital length of stay decreased	Waiting time improved
	PhilHealth still used paper-based claims management	Data gathering was existent; however, intensified and modernized effort was needed
	Absence of an integrated curative and preventive network	Increased use of rural health units, decreased use of private clinics
	Low sponsored program PhilHealth utilization rate	Treatment seeking attitude improved among households
Retrogressive	Introduction of health technology assessment (HTA) by PhilHealth (in identifying priority problems on the use of medical technologies needing systematic assessment)	Health technology assessment (HTA) was yet to be fully established
	Decentralization of health care services (fragmented health service delivery)	Fragmented nature of health financing, devolved structure of service delivery, and mixed public–private health system posed immense challenges in monitoring health sector performance
	Rising non-communicable diseases (NCDs)	Obesogenic environment; life-style-related health problems
	High cost of accessing health service	Healthcare provision tended to be either underprovided or overprovided, and costly
	Low level financial protection	PhilHealth insurance claims stagnated at 33%
	High out-of-pocket (OOP) payments	High OOP payments
	Weak health information system/governance	Adherence to clinical practice guidelines were loose
		Patient safety data was lacking
	Absence/lack of access of private sector data	Health data acquisition was still restricted (private sector, public sector, PhilHealth)
	Uneven distribution of PhilHealth accredited providers (35% of doctors are in NCR)	Uneven distribution of health staff across the country (concentrated in NCR)
	Uneven distribution of health facilities and beds across the country	Uneven distribution of health facility across the country (concentrated in NCR)
		Challenges in regard to hospital bed availability

Green = Feature; Orange = Challenge; Progressive classification: (a) Challenge changed to Feature; (b) Feature retained as Feature; Retrogressive classification: (a) Feature changed to Challenge; (b) Challenge retained as Challenge. NCR = National Capital Region.

## Supplementary Materials

The following are available online at https://www.mdpi.com/2227-9032/7/4/116/s1, Table S1: Master dataset of the annual health system performance-related variables, Table S2: Variable description and related source.
