# Supplementary material for: Developmental Changes in the Philippine Health System: Accomplishments, Successes and Challenges
Source: Healthcare (Basel). 2019 Oct 14;7(4):116. doi: 10.3390/healthcare7040116 (PMC6955948; doi:10.3390/healthcare7040116)
Supplement: Supplementary file 1 [file healthcare-07-00116-s001.pdf]

Supplementary Materials:

Table S1. Master dataset of the annual health system performance-related variables.

Year	Lifeexp	Mmr	Imr	Hiv	Tb	Wasting	Overweight	The	Sin	Hivexp
1997	66.668	119	31.4	200	NA	NA	NA	NA	NA	NA
1998	66.851	122	30.7	200	NA	NA	1.9	NA	NA	NA
1999	67.019	117	30	500	NA	NA	NA	NA	23208	NA
2000	67.17	124	29.4	500	590	NA	NA	3.154814	24449	NA
2001	67.308	128	28.8	500	579	NA	NA	2.947058	27163	NA
2002	67.434	127	28.2	500	569	NA	NA	2.733304	28155	NA
2003	67.555	125	27.6	610	559	1.6	2.4	3.191438	28048	NA
2004	67.67	126	27	770	549	NA	NA	3.167925	32355	NA
2005	67.783	127	26.5	980	539	NA	NA	3.900419	33263	10.31
2006	67.892	127	26.1	1200	530	NA	NA	3.94585	37619	11.42
2007	68	128	25.6	1600	520	NA	NA	3.919541	32557	7.68
2008	68.105	129	25.2	2100	524	2	3.3	4.027922	38667	11.15
2009	68.211	131	24.9	3600	528	NA	NA	4.353532	34002	16.99
2010	68.32	129	24.5	4400	531	NA	NA	4.312762	44516	17.87
2011	68.433	127	24.2	4600	535	2.4	4.3	4.206093	36473	18.67
2012	68.553	126	23.9	5400	539	NA	NA	4.374653	46216	14.48
2013	68.68	121	23.6	6600	543	NA	5	4.463811	100463	15.78
2014	68.813	117	23.3	8200	546	NA	NA	4.188945	116060	NA
2015	68.951	114	23	9600	550	NA	3.9	4.413516	139602	27.87
2016	69.094	NA	22.7	11000	554	NA	NA	NA	NA	NA
2017	NA	NA	22.2	12000	NA	NA	NA	NA	NA	43
2018	NA	NA	NA	NA		NA	NA	NA	NA	NA

Table S2. Variable description and related source.

Variable	Description	Source
year	year	
lifeexp	life expectancy at birth	World Bank [1]
mmr	maternal mortality rate (per 100,000 live births)	World Bank [2]
imr	Mortality rate, infant (per 1,000 live births)	World Bank [3]
hiv	HIV incidence	Department of Health—Philippines [4]
tb	Incidence of tuberculosis (per 100,000 people)	World Bank [5]
wasting	Prevalence of severe wasting, weight for height (% of children under 5)	World Bank [6]
overweight	Prevalence of overweight, weight for height (% of children under 5)	World Bank [7]
the	Current health expenditure (% of GDP)	World Bank [8]
sin	Inflation-adjusted tobacco excise tax (Philippine peso, in millions)	OECD [9]
hivexp	Inflation-adjusted HIV expenditure (USD millions)	AIDS Datahub [10]

References

1. World Bank. Life Expectancy at birth. Availabe online: https://data.worldbank.org/indicator/SP.DYN.LE00.IN?locations=PH (accessed on 24 August 2019).
2. World Bank. Maternal Mortality ratio (per 100,000 live births). Availabe online: https://data.worldbank.org/indicator/sh.sta.mmrt (accessed on 24 August 2019).
3. World Bank. Mortality rate, infant (per 1,000 live births). Availabe online: https://data.worldbank.org/indicator/SP.DYN.IMRT.IN (accessed on 24 August 2019).
4. Department of Health—Philippines. *HIV/AIDS & ART Registry of the Philippines*; Department of Health—Philippines: Manila, Philippines, 2019.
5. World Bank. Incidence of tuberculosis (per 100,000 people). Availabe online: https://data.worldbank.org/indicator/SH.TBS.INCD (accessed on 24 August 2019).
6. World Bank. Prevalence of severe wasting, weight for height (% of children under 5). Availabe online: https://data.worldbank.org/indicator/SH.SVR.WAST.ZS?view=chart (accessed on 24 August 2019).
7. World Bank. Prevalence of overweight, weight for height (% of children under 5). Availabe online: https://data.worldbank.org/indicator/SH.STA.OWGH.ZS?view=chart (accessed on 24 August 2019).
8. World Bank. Current health expenditure (% GDP). Availabe online: https://data.worldbank.org/indicator/SH.XPD.CHEX.GD.ZS (accessed on 24 August 2019).
9. OECD. Details of Tax Revenue—Philippines. Availabe online: https://stats.oecd.org/Index.aspx?DataSetCode=REVPHL (accessed on 24 August 2019).
10. AIDS Datahub. AIDS Financing Availabe online: https://www.aidsdatahub.org/Country-Profiles/Philippines (accessed on 24 August 2019).
